# Decay Resistance of Surface Carbonized Wood

**DOI:** 10.3390/ma15238410

**Published:** 2022-11-25

**Authors:** Maija Kymäläinen, Tiina Belt, Hanna Seppäläinen, Lauri Rautkari

**Affiliations:** 1 Department of Bioproducts and Biotechnology, School of Chemical Engineering, Aalto University, Aalto, P.O. Box 16300, FI-00790 Espoo, Finland; 2 Production Systems, Natural Resources Institute Finland, Viikinkaari 9, FI-00790 Helsinki, Finland

**Keywords:** basidiomycetes, brown rot, char, surface modification, white rot, wood

## Abstract

Surface carbonization, or charring, of wood is a one-sided modification method primarily intended for protection of exterior cladding boards. The heavily degraded surface acts as a barrier layer shielding the interior from environmental stresses, and as such acts as an organic coating. To test the durability of surfaces created in this manner, unmodified, contact charred, and flame charred spruce and birch samples were exposed to the brown rot fungus *Coniophora puteana* and white rot fungus *Trametes versicolor* for a period of nine weeks. All sides of the samples except the modified surfaces were sealed to investigate the protective effect of the surface. Mass losses were greatest for unmodified references (up to 60% and 56% for birch and spruce, respectively) and smallest for contact charred samples (up to 23% and 32%). The wood below the modified surfaces showed chemical changes typical of brown rot and simultaneous white rot. The measured glucosamine content revealed fungal biomass in both the modified surface as well as the layers beneath. According to the recorded values, the fungal biomass increased below the surface and was higher for flame charred samples in comparison to contact charred ones. This is likely due to the more intact, plasticized surface and the thicker thermally modified transition zone that restricts fungal growth more effectively in contact charred samples in comparison to the porous, cracked flame charred samples. Scanning electron microscope images verified the results by revealing fungal hyphae in all inspected wood types and species.

## 1. Introduction

Surface carbonization, or charring, of wooden boards is a modification method that aims to improve the durability and weatherability of claddings. The technique is originally based on the Japanese tradition of burning sugi (*Cryptomeria japonica*) wood boards (yakisugi = burned Japanese cedar) to gain fire and decay resistant exteriors for a low cost [1]. Nowadays, there are several producers in the USA and Europe, and the interest in this ecological, natural, and aesthetic surface modification is increasing [2]. The basic process of surface carbonization is quite simple and relies on high temperatures that degrade the surface to a carbonaceous residue, or charcoal. The techniques include the traditional “free” flame charring, where the boards are set on fire by ignition flame [3,4], and the more controlled gas flame charring, where boards are either charred by a handheld torch or conveyed beneath static torches [5,6,7]. Not yet commercialized, the contact charring technique relies on the use of a heated plate in tight contact with the modified wood surface, providing a highly repeatable and controlled result [6,7,8,9,10,11,12,13,14].

The flame charred surface is chemically rather inert, built of aromatized compounds and a mix of amorphous and graphitized carbon [6]. A carbonized wood surface entails highly condensed aromatic ring structures, but the exact composition depends on wood species, wood type, and, importantly, process conditions. Thus, a general charcoal may be described as a heterogeneous mixture of partially degraded and heat-altered biomacromolecules [15]. It is nevertheless known that an increase in modification temperature will promote the change from amorphous to a more ordered graphitized structure, as was also seen in surface (flame) charred woods [6]. This kind of surface was shown to be quite recalcitrant against weathering and is also hypothesized to be a poor substrate for decay fungi. For example, Prosen et al. [16] reported the aromatic species formed in pyrolysis to be inhibitory to the growth of *Phanerochaete chrysosporium*. The purpose of the char surface is to limit, if not entirely inhibit, biological agents causing structural decay via depletion of carbohydrates as well as formation of modified lignin and other degradation products.

Research on the durability of carbonized wood surfaces is still scant and the results are somewhat inconsistent. Okamura et al. [1] investigated the properties of yaki sugi manufactured following the traditional method and exposed samples to *Pleurotus* on agar. Mass loss determinations showed a higher degradation in comparison to unmodified sugi references. Machová et al. [12] used the contact charring technique on beech and concluded that the modification decreased resistance to *Coniophora puteana* but increased the resistance towards *Trametes versicolor*. The samples were exposed only through the modified surface with other sides sealed. In soil contact, the char tends to decrease service life, as was reported for Douglas fir fence posts by Graham [17]. The porosity increases with increasing modification temperature and thus promotes capillary uptake of moisture [18]. In a soil block assay, Hasburgh et al. [2] examined commercial surface carbonized wood samples using *Gloeophyllum trabeum* and *T. versicolor*. As a result, cypress showed improvement while other woods (acetylated and furfurylated woods, black walnut, hemlock, and white oak) did not benefit from surface charring. However, it was not clear whether only the surface had been exposed. As the intended use of surface carbonized wood is for building façades and claddings, no ground contact is expected and the potential exposure to fungal spores takes place only through the surface. In comparison to a flame charred surface, the contact charred surface is created by using lower temperatures and is therefore not as far degraded. The resulting surface has few cracks and a thick thermally modified transition layer that may further restrict fungal activity. The final properties, including the decay resistance, of charred wood are dependent on wood species and exposure conditions. However, the interactions are complex and understanding of specific cell wall changes responsible for protective effects in thermally degraded wood is still lacking [19].

The aim of this study was to investigate the level of protection provided by the charred surface against common decaying fungi. It has previously been shown that a flame charred surface is rather inert against weathering damage, thus whether it would also provide protection against decay was further examined. Contact charring of wood differs from flame charring in terms of exposure temperature and duration, and the resulting product presents a smooth, hard surface with highly improved sorption properties [7]. For this reason, contact and flame charred wood surfaces were compared. Lastly, previous results have shown that surface carbonized birch wood may have favorable properties in outside use, thus spruce and birch wood were both included in this study.

## 2. Materials and Methods

### 2.1. Sample Preparation

The materials used in this study were sapwood of softwood Norway spruce (*Picea abies* (L.) Karst.) and hardwood silver birch (*Betula pendula* Roth.) sourced from Southern Finland. Two methods were used to modify the two wood species: a contact charring technique using a heated stainless-steel plate and a weight of 16 kg placed on top of the charring sample to avoid cupping, and a flame charring technique using a butane gas torch applied from an even height along the board. The charring was always implemented on the pith side of planed samples. The contact charred samples were cut to 100 mm × 100 mm (tangential × radial) due to dimensions of the hot plate. The samples were exposed to a temperature of 320 °C for a duration of 30 min. This regime was chosen based on previous tests [6,7] to avoid surface cracking and create a thick transition layer. The flame charring was implemented outdoors, moving the torch slowly along the length of a one-meter board. The charring was continued until the formation of a consistent crack pattern, which took about 2.5 to 3 min per board.

After modification, final specimens were cut in dimensions of 1 mm × 1 mm × 1 (±1) mm using a small tabletop circular saw. Specimens with the same dimensions were also cut from unmodified spruce and birch to act as references. Only specimens without visible defects were chosen, avoiding the edges of the charred samples due to unevenness of char layer thickness. The specimens were oven dried (OD) at 103 °C before sealing all sides except the modified surface with sanitary grade silicone (Ardex SE, Witten, Germany), then oven dried again to determine the OD mass of both the sample and the silicone for further mass determination. The specimens were finally sterilized with gamma irradiation (Scandinavian Clinics Estonia OÜ, Estonia) using a 25–50 kGv dose before the decay test. The codes used to describe samples are shown in Table 1.

### 2.2. Decay Test

**Figure 1:**
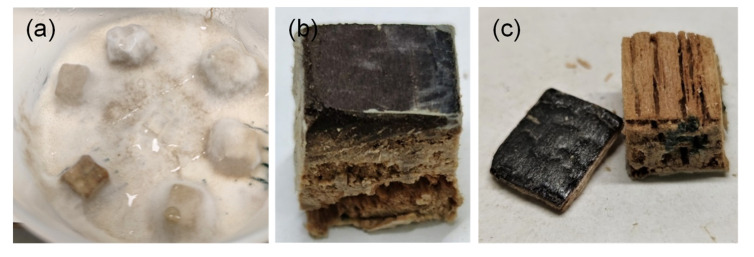
(**a**) Spruce references on *C. puteana* prior to cleaning and mass loss determination; (**b**) sample SC-CP after silicone removal: the surface is intact but beneath the modified layers the wood has been degraded; (**c**) sample SF-CP after silicone removal: the wood beneath the char and transition zones has been consumed.

The decay test was conducted in 112 mm diameter screw-cap jars containing 50 mL of 2% malt extract agar. To ensure exchange of vapors, a hole was drilled into the lid and plugged with cotton. Each jar was inoculated with one plug of mycelium from the growing edges of *C. puteana* (Schum. ex Fries) Karst. (strain BAM Ebw. 15) or *T. versicolor* (L.) Lloyd (strain PRL 572) stock cultures maintained on 2% malt extract agar. After 13 days of incubation, the sterilized wood specimens were placed on the colonized agar with the unsealed surface of the samples facing downward. A thin plastic mesh was placed between the samples and the agar to prevent capillary water sorption from the medium. Each jar received one replicate specimen of every type (Figure 1a). A total of 10 replicates of each sample type were used per fungal species. The vessels were placed in a climate chamber (Rumed 5100, Rubarth Apparate GmbH, Laatzen, Germany) at 85% RH, 20 °C. At 9 weeks the jars were taken out and the decay test terminated. The samples were cleaned to remove all adhering mycelium and weighed to determine their wet mass, after which they were first dried at ambient humidity overnight before oven-drying at 103 °C for 24 h to determine their dry mass. The mass loss was calculated according to Equation (1):

(1) $$\mathrm{Mass}\;\mathrm{loss}\;(\%)=((Mass_{0}-Mass_{sil})-\left(Mass_{1}-Mass_{sil}\right))/\left(Mass_{0}-Mass_{sil}\right)*100$$

where *Mass*_0_ is the OD mass before the decay experiment, *Mass_sil_* is the mass of silicone, and *Mass*_1_ is the OD mass of the decayed specimen.

### 2.3. Elemental Analysis

The elemental compositions of modified sample surfaces were analyzed with FlashSmart EA CHNS/O (Thermo Fischer Scientific, Waltham, MA, USA). Samples were ground with a Wiley mill (Wiley Mini Mill 475-A, Thomas Scientific, Swedesboro, NJ, USA) to pass through a 20-mesh sieve. A 2–3 mg sample was first combusted, followed by flow through the catalyst and copper reduction phase with the carrier gas helium. A GC column separated the combustion gases, which were detected by a thermal conductive detector (TCD). The analysis was performed in triplicate.

### 2.4. Determination of Chemical Composition

Samples were taken from the surface (0 mm) and at 2 mm depth from the surface of the specimens to determine the chemical composition before and after fungal degradation. Prior to the decay test, the surfaces of interest were extracted from larger wood pieces. After the decay test the analyzed sets were formed by combining 9 replicate wood blocks (one from each jar), with the surface mycelium and the silicone removed (Figure 1b,c). The OD samples were ground to a powder passing through a 20- or 30-mesh sieve using a Wiley mill (Wiley Mini Mill 475-A, Swedesboro, NJ, USA). The larger mesh was applied for unmodified samples and the smaller for modified. Flame charred surfaces were crushed by hand using a mortar to prevent excessive dusting. The samples were Soxhlet extracted with acetone according to SCAN-CM 49:03 [20]. Structural carbohydrates and acid soluble and Klason lignin were then determined by acid hydrolysis using a miniaturized version of NREL/TP-510-42618 [21]. Wood powders (approx. 0.04 g dry mass) were first pre-hydrolyzed with 0.4 mL of 72% (*w*/*w*) sulphuric acid for 60 min at 30 °C, after which 11.2 mL of MilliQ water (Synergy ultraviolet purification system, Millipore SAS, Molsheim, France) was added. The hydrolysis was completed by autoclaving at 121 °C for 60 min and the hydrolysates were filtered through glass crucibles. After dilution with MilliQ water, the monosaccharide composition of the hydrolysates was analyzed by High-Performance Anion-Exchange Chromatography with Pulsed Amperometric Detection (Dionex ICS-3000 HPAEC-PAD, Thermo-Fischer Scientific, Waltham, MA, USA) using a CarboPac PA20 column and deionized water as the eluent at 0.37 mL/min. Klason lignin was determined by drying the glass crucibles overnight at 105 °C and then weighing the insoluble hydrolysis residue, while acid soluble lignin was determined from the hydrolysis liquid by UV-Vis spectrometry (Shimadzu UV-2550, Shimadzu, Kyoto, Japan) using a wavelength of 205 nm. All analyses were made in duplicates. In addition to the monosaccharides derived from wood structural carbohydrates, the glucosamine content of the hydrolysates was also analyzed. Glucosamine is a constituent of fungal cell walls and can be used as a quantitative measure of fungal biomass [22]. Glucosamine was analyzed simultaneously with the other monosaccharides using HPAEC-PAD, with quantitation performed using a series of external glucosamine standards.

### 2.5. Microscopy

Decayed samples and unexposed references were observed with a scanning electron microscope (SEM; Carl Zeiss Microscopy GmbH, Jena, Germany) to detect signs of fungal growth. The aim was to more closely investigate whether the fungus would simply grow through the surface or additionally cause structural degradation. Only one randomly selected block from each jar was investigated, while the remaining 9 were taken for chemical analysis. The blocks were used as is, but a small piece of the surface was cut with a razor blade to allow investigation also in the transverse direction. The specimens were sputter coated with a 5 nm layer of Au/Pd. Due to image quality issues, some samples were redried and recoated with another 5 nm of Au/Pd before repeating the imaging procedures. The images were recorded with an acceleration voltage of 3 to 5 kV.

## 3. Results

### 3.1. Elemental and Chemical Analysis of Undegraded Spruce and Birch

The increasing modification temperature caused a major change in the carbon and hydrogen contents of both spruce and birch (Table 2). The increase in C and decrease in H content was nearly linear compared to reference. Sulphur and nitrogen were below detection limits and therefore not considered further.

**Figure 2:**
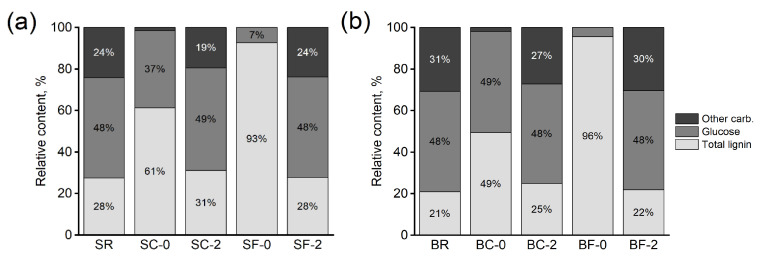
Relative content of total lignin (acid soluble + Klason), glucose and other carbohydrates in undecayed (**a**) spruce (SR) and (**b**) birch (BR) references and modified samples measured at 0 or 2 mm depth from the surface. R = unmodified; C = contact charred; F = flame charred; S = spruce; B = birch.

Supporting the elemental analysis results, both the undegraded spruce and birch samples showed an increasing lignin content with increasing severity of the modification (Figure 2; Table S1). The value presented is a combination of relative amounts of solid and acid soluble lignin. The relative lignin content of unmodified spruce (SR) was about 27%, that of contact charred spruce (SC) 61%, and that of flame charred spruce (SF) almost 93% (Figure 2a). During surface carbonization, birch underwent similar changes to spruce (Figure 2b). The original lignin content of about 21% increased to 50% and to 96% on BC and BF, respectively. This indicates a very heavily carbonized surface on BF. The amount of holocellulose was higher on BC than on SC (about 50 % to 39%, respectively), which shows that spruce was slightly further pyrolyzed within this regime. The samples taken from 2 mm beneath the surface showed a slightly higher content of cellulose and lignin on SC/BC in comparison to SF/BF, revealing the extent of the thermally modified transition zone that continues deeper beneath the surface on contact charred than flame charred samples. The references were practically identical at 0 and 2 mm depth from the surface and very similar to both SF and BF at 2 mm depth. The results for total lignin content are in line with the elemental analysis results. In an unmodified wood sample, the carbon is mostly found in lignin and carbohydrates. As the relative amount of carbohydrates decreases due to the thermal degradation process, the share of lignin increases. In pyrolysis, lignin preferentially forms char [23,24]. Additionally, other lignin-like aromatic compounds are formed, further increasing the relative lignin content.

### 3.2. Mass Loss Due to Decay

**Figure 3:**
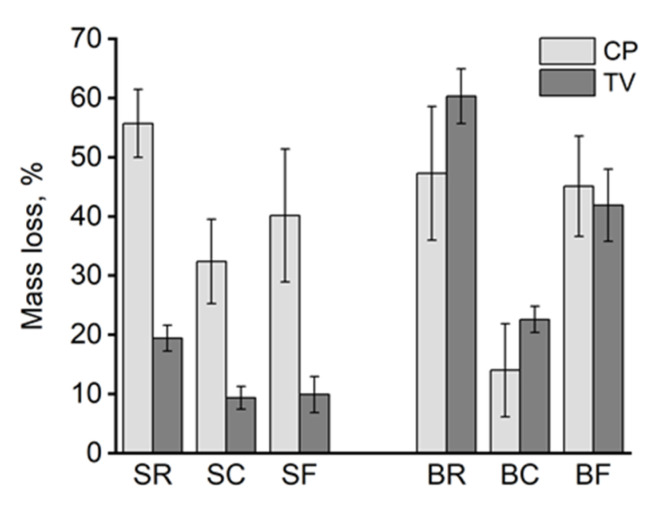
Mass loss of spruce and birch (%) samples exposed to decay fungi *C. puteana* (CP) and *T. versicolor* (TV). Error bars for 95% confidence interval. R = unmodified; C = contact charred; F = flame charred; S = spruce; B = birch.

As expected, the highest average mass losses were found in the unmodified spruce and birch samples (Figure 3). The unmodified samples had turned from hard to soft and spongy. The brown rot CP caused a higher mass loss on unmodified spruce samples than the white rot TV, while TV caused a slightly higher mass loss on unmodified birch samples than CP. The difference in mass loss between the two fungi on reference samples was statistically significant only on spruce. On modified spruce samples, losses caused by TV were moderate (below 10% for SC and SF), with a 50% reduction in comparison to the unmodified samples (SR). Mass losses caused by CP were smallest for contact charred samples on both spruce and birch. The losses were 42% (SC) and 70% (BC) lower in comparison to the unmodified references of the respective wood species. The same trend was also observed for BC–TV, with a 63% (BC) lower mass loss compared to the unmodified reference. The mass losses caused by CP and TV differed significantly from each other within every modification, but within the TV-series, mass losses do not differ significantly between contact charring and flame charring modifications. On birch, only BC mass losses showed significant differences between the two fungi, i.e., there is no difference between the BR and BF treatments in terms of mass loss at a confidence level of 95%.

### 3.3. Chemical Analysis of Decayed Spruce and Birch

**Figure 4:**
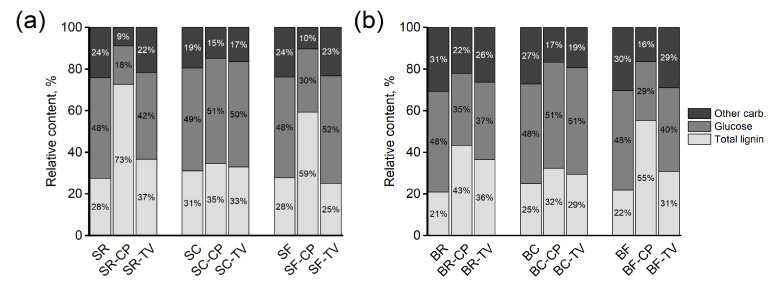
Relative content of total lignin (acid soluble + Klason), glucose and other carbohydrates in (**a**) spruce and (**b**) birch samples exposed to decay fungi *C. puteana* (CP) and *T. versicolor* (TV). Values are averages of duplicate analyses on pooled samples. R = unmodified; C = contact charred; F = flame charred; S = spruce; B = birch.

The chemical composition of the samples was further investigated from the surface (0 mm depth) and beneath the charred surface (2 mm depth) to understand the extent of decay (Figure 4). Comparison of results is complicated by the modifications, as the references are bulk samples and the contact and flame charred ones are slices cut from the surface or the area beneath. It was also seen that the 0 mm results of flame charred samples are unreliable due to loss of surface material as some of the char had flaked off and become embedded in the agar media used in the decay test. Thus, only results from 2 mm depth are presented. All samples exposed to CP showed a decrease in carbohydrate content and an increase in lignin content, indicating depletion of holocellulose. The changes were more pronounced in SR, SF, BR, and BF, in agreement with the high mass losses seen in these samples (Figure 3). The contact charred samples (SC and BC) showed more limited changes in composition. The samples exposed to TV revealed only slight compositional changes, as could be expected after degradation by simultaneous white rot. All samples exposed to TV showed slight losses in non-glucose sugars, while the more extensively degraded samples (BF, BR) also showed a loss in glucose and an increase in lignin content. The SF samples did not differ substantially from the SR samples, meaning that, at 2 mm depth from the surface, the flame charred wood behaves as unmodified wood.

### 3.4. Quantification of Fungal Biomass

**Figure 5:**
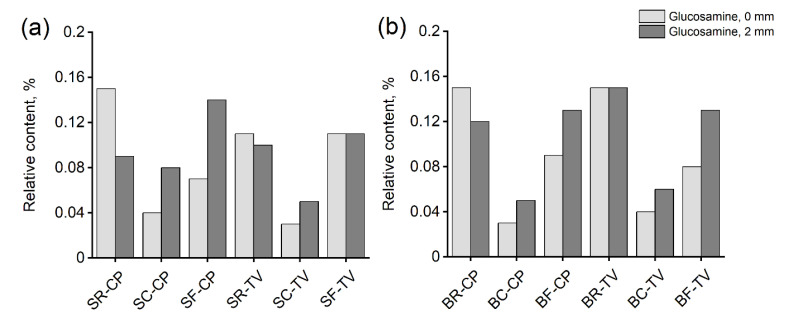
Relative content of glucosamine from the surface (0 mm) and 2 mm beneath the surface in (**a**) spruce and (**b**) birch samples exposed to *C. puteana* (CP) and *T. versicolor* (TV). Values are averages of duplicate analyses on pooled samples. R = unmodified; C = contact charred; F = flame charred; S = spruce; B = birch.

Glucosamine content can be used as an indicator of fungal biomass quantity (Jones and Worral 1995). The relative content of glucosamine was the highest for the degraded unmodified references and followed the trend of mass losses (Figure 5). The content was determined from the surface at 0 mm and beneath the surface at 2 mm to investigate the amount of biomass in the modified layer and in the material beneath it. From the figure, it is evident that while there was detectable glucosamine within both layers, the relative amount generally increased at 2 mm in the modified samples, i.e., the amount of fungal biomass was higher beneath the modified surface than at the surface. However, as some char was lost in handling of the flame charred samples, the results for SF and BF are not directly comparable. It is assumed, however, that the observed trend holds true for reference and contact charred samples.

### 3.5. Microscopical Examination

**Figure 6:**
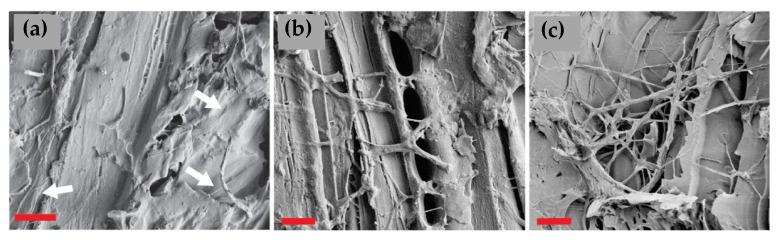
Surfaces of (**a**) SC-CP, with arrows pointing to thin hyphae of *C. puteana*; (**b**) SC–TV and (**c**) SF–TV, both showing thick growths of *T. versicolor*. Imaged at 1000× magnification, bar = 10 µm.

**Figure 7:**
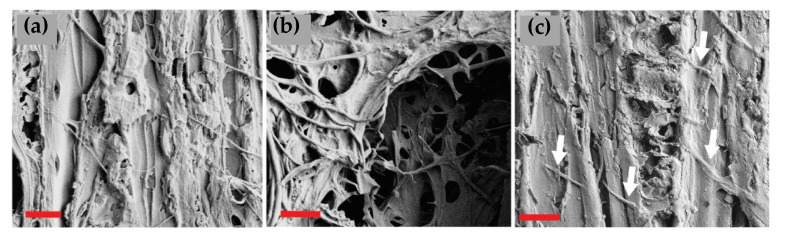
Surfaces of (**a**) BC-CP showing both thin hyphae of *C. puteana* and cell wall degradation visible as ragged structures; (**b**) abundant growth of *C. puteana* on BF–CP; (**c**) BF–TV imaged with arrows pointing to hyphae of *T. versicolor*. Imaged at 1000× magnification, bar = 10 µm.

**Figure 8:**
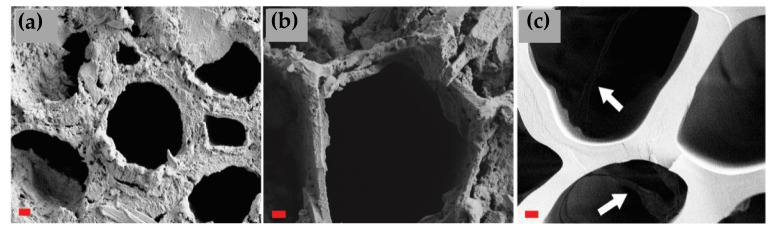
Modified layer cross-sections of (**a**) BC–TV; (**b**) SC-CP; (**c**) SF–CP with arrows pointing to faintly visible hyphae of *C. puteana* inside the lumens. Imaged at 2000×, bar = 2 µm.

The unmodified, undegraded reference samples (Figures S1 and S2 in Supplementary Materials) of both spruce and birch showed a typical structure with some drying-induced cracking in the fiber direction. The contact charred samples were plasticized with no loose fibers but revealed some cell wall damages induced by drying, handling, and the surface modification. Flame charring transformed the surface to a charcoal with no distinct cell wall layers. The cracks induced by shrinking and thermal degradation had sharp edges due to the friable nature of charcoal in comparison to the fibrous virgin wood. The degraded samples hosted thick “mats” of hyphae, especially in the surface cracks of flame charred samples (Figure S3 in Supplementary Materials). Fungal hyphae were detected in almost all investigated samples (Figure 6 and Figure 7). The hyphae of *T. versicolor* were slightly thicker than those of *C. puteana* and therefore easier to detect. As expected, the damage was worst in the reference samples, with extensively degraded cell wall structures (Figure S4). Some signs of degradation were detected on the tangential surfaces of contact charred samples, but the cross-sections (Figure 8a,b) revealed more structural damage. The flame charred samples, imaged from 0 mm depth, presented a slightly distorted structure in their native state, so providing exact evidence of the contribution of fungi is more complicated. However, when looking at the cross-section imaged within the char layer, no cell wall degradation was seen. Instead, hyphae were detected inside the lumens (white arrows on Figure 8c).

## 4. Discussion

Spruce and birch wood surfaces were modified using the flame charring and contact charring methods. Elemental analysis (Table 2) revealed a large increase in the carbon content of the surfaces, while compositional analysis following acid hydrolysis showed a decrease in carbohydrate content and an increase in Klason lignin content (Figure 2). The contact charred surfaces were almost entirely depleted of hemicelluloses but still contained a large fraction of cellulose, while the flame charred surfaces were almost entirely devoid of all carbohydrates and consisted primarily of Klason lignin. The Klason lignin detected is not all lignin, but insoluble char formed also from hemicelluloses and, to a small degree, from cellulose. Lignin preferably forms char, but hemicelluloses may contribute over 20% of the yield (beech, above 700 °C; [25]). Under the modified surface, the composition of the samples resembled unmodified wood with a slight increase in lignin content, particularly in the contact charred samples. Using the flame charring technique, the surface char layer is always about 1–2 mm thick [1,3,4]. This depends both on the poor thermal conductivity of the charcoal that hinders the temperature increase within the transition zone, as well as on the exposure time, as charring rate is presumed constant. The transition zone in a flame charred wood sample is a couple of cell layers thick [26], while in a contact charred sample the thickness may exceed several millimeters depending on modification time [14]. For this reason, the contact charred samples are modified deeper and exhibit a slightly higher lignin and lower hemicellulose content at 2 mm depth in comparison to flame charred samples. As contact charring is not yet a commercial technique, comparative results are scarce. Machová et al. [12] reported a shift of carbohydrates towards the surface on carbonized specimens, but the modification durations used were very short. This may lead to transfer of substances due to temperature increase, followed by retention on surface due to partial, suspended evaporation as the heating is terminated. A longer modification time promotes thermal degradation of wood components [27,28], but it is also to be noted that a longer carbonization time reduces the pyrolysis rate as the char layer thickens, thus slowing down the reactions [29].

The decay resistance of the modified samples was tested against brown rot (CP) and white rot (TV) by exposing only the modified surface. The results showed that both surface charring techniques reduced the mass losses caused by CP and TV on both spruce and birch relative to the untreated references (Figure 3), with contact charring (SC and BC) proving more effective at protecting wood than flame charring (SF and BF) on both wood species and against both fungi. There was no practical difference between the mass losses of CP degraded SR and BR, or SF and BF. To determine the extent to which degradation took place on the modified surface and in the layers beneath it, the chemical composition of the samples was analyzed using acid hydrolysis. Because of problems with analyzing the decayed flame charred surfaces (i.e., char flaking off while removing samples from the test jars) it was not possible to say exactly how much of the charred wood was consumed and altered by the fungi. Nevertheless, the recorded SEM images showed evidence of cell wall degradation taking place in the contact charred surfaces (Figure 7 and Figure 8), whereas the flame charred surfaces were more intact. The samples collected from beneath the charred surface (Figure 4) showed chemical changes due to decay that were typical of brown rot (CP) and simultaneous white rot (TV), confirming that the fungi degraded the material beneath the modified layer. The changes were generally more pronounced in samples with high mass losses, which demonstrates the protective effect of the surface modifications, particularly contact charring.

In addition to the wood cell wall structural constituents, the glucosamine contents of the samples were also measured to determine the quantity of fungal biomass in both the charred surface and the layers beneath it. The results (Figure 5) showed that the glucosamine contents were lower in the contact charred samples than in the flame charred or reference samples for both fungi on both wood materials, in agreement with the mass loss and chemical composition results. The glucosamine contents of references were higher on the surface (0 mm), while the reverse was true for modified samples (higher glucosamine content at 2 mm). Substantial biomass was still present in the modified layer, confirming that the fungi are capable of growing within the modified surface. Due to thermal degradation, volatilization, and the reorganization of structures, the readily available nutrients are severely depleted in char. However, utilizable components such as levoglucosan and fatty acids often remain [30], and degradation of high molecular weight organic molecules in hardwood charcoal has been reported [31]. The presence of hyphae on the surfaces of our samples was also confirmed microscopically (Figure 6, Figure 7 and Figure 8), although the investigation revealed that they were most concentrated along lines of structural weakness. It seems plausible the fungi mainly used the char pores and cracks to enter the unmodified wood beneath. Similar conclusions were drawn by Ascough et al. [32], who subjected carbonized pine to *Pleurotus pulmonarius* and *Trametes (Coriolus) versicolor*. Cracks were abundant on the surface of the flame charred samples, which may at least partially explain the lower decay resistance of BF and SF in comparison to BC and SC. These cracks are results of the sharp temperature and moisture gradients, as well as the anisotropic shrinkage of wood that leads to dimensional instability. The cracks run through the char layer into the transition zone and provide useful pathways for the fungal hyphae. On the contrary, the contact charred surfaces were manufactured at conditions that limit extensive cracking. The carbonization process begins at around 300 °C [33], which is roughly the point where crystalline cellulose begins to break down [34,35]. Using our device, the surface began to crack at approximately 330 °C, practically regardless of exposure duration. Even though the fungi were found capable of growing through the modified layer to degrade the wood material beneath it, the modifications were able to reduce the mass loss caused by the fungi.

The protective effect of surface carbonization is likely due to a combination of mechanisms, including limited nutrient availability. The modified surfaces have a reduced concentration of carbohydrates, particularly hemicellulose, which is the first major component to be attacked by fungi [36,37]. Hemicelluloses are the most thermally labile components in wood and will be severely depleted in thermal modification, and even further in carbonization. The loss of hemicelluloses is, however, likely to have only a limited protective effect as the fungi will utilize cellulose in the absence of hemicelluloses (reviewed by Ringman et al. [38]). Charcoal manufactured at a relatively low temperature of 300 and 400 °C was stated to form a nutrient source not readily available for fungi [32]. When the point of over 90% of insoluble compounds is reached, as in the flame charred samples, it is presumed that the rate of fungal degradation is likely to be very slow. However, as was seen in the SEM images, the hyphae move through the voids and eventually reach the intact unmodified substrate underneath and begin to degrade it if the conditions continue to support decay.

In addition to reduced nutrient availability, thermal degradation and loss of hemicelluloses reduce the hygroscopicity of the surface layer similarly to heat treated wood [7,8,39]. Decay of wood that is not in contact with the ground is often related to entrapment of rainwater [40]. In building façades, the risk is normally quite small, if proper design and installation precautions are taken into consideration. However, driving rain and uneven wood surfaces may create water traps. The char layer formed by flame charring is porous and soft and readily absorbs water [7]. The porosity and high permeability also aid in fast water release, but only if the surface is allowed to dry out. The char surface may also be susceptible to accumulation of debris such as pollen, that with moisture creates a good substrate for micro-organisms [37]. However, despite cracking and microscopic debris, mold growth was inhibited on the charred surfaces exposed to natural weathering [6], indicating difficulties in spore germination. In contrast to the flame charred surfaces, contact charring greatly improves the contact angle [8,9] and modified samples exhibit increased hydrophobicity towards water in both liquid and vapor form [7,9].

Finally, the migration of extractives to the surface may also play a role in the decay resistance of surface carbonized wood. The extractives are generally volatile and escape early in combustion. However, in the (long duration) contact charring process, the extractives move towards the surface but are at least partly unable to vaporize due to tight contact between the wood piece and the hot surface. Flaming combustion is also avoided due to this reason, at least up to 500 °C (the operational limit for out device). In a severely restricted airspace, the volatilized extractives will accumulate and boil and then cool on the surface, creating a smooth, glossy and hard surface similar to surface densified wood [41,42]. This, in combination with the smoother, harder surface and the thick transition zone, contributed to lower observed mass loss in SC and BC.

The results presented here indicate that surface charring can improve the resistance of spruce and birch wood to degradation by brown rot and white rot fungi. The protection provided by the modifications was moderate in the agar block test utilized in this study, but it should be noted that the surface charred wood is likely to be utilized in applications where the conditions are less optimal for decay.

## 5. Conclusions

Norway spruce and silver birch were surface modified by one-sided carbonization using the flame charring and contact charring techniques. The absence of surface cracks, as well as the very hydrophobic surface on contact charred woods, seems to efficiently limit fungal activity, based on mass loss measurements, chemical analysis, and estimates of fungal biomass in the samples. A flame charred surface is further thermally degraded, but the cracked, porous substance provides access for fungal hyphae into the underlying unmodified layers of the surface carbonized wood. The presence of hyphae inside the lumina of flame charred wood was verified, whereas clear evidence of cell wall degradation was not seen. In terms of wood species, the initial differences in composition between the investigated soft- and hardwood seem to have very little effect on mass loss caused by *C. puteana*, whereas *T. versicolor* clearly favored birch, causing higher mass losses also on the modified samples. However, the measured glucosamine contents on the degraded samples did not differ much between the two species and fungi. The ability of fungal spores to germinate on charred surfaces in a natural setting is yet to be determined.

## Figures and Tables

**Table 1:** Coding for species and modification.

Species	Modification	Code
Spruce	Unmodified	SR
	Contact charred	SC
	Flame charred	SF
Birch	Unmodified	BR
	Contact charred	BC
	Flame charred	BF

**Table 2:** Elemental composition of unmodified and modified spruce and birch surfaces. Standard deviation (n = 3) in parenthesis.

Species	Sample	C, %	H, %
Spruce	SR	46.9 (0.2)	5.9 (0.0)
	SC	57.2 (0.3)	5.2 (0.1)
	SF	70.5 (0.2)	3.6 (0.1)
Birch	BR	45.8 (0.1)	5.8 (0.0)
	BC	54.2 (0.2)	5.3 (0.0)
	BF	71.8 (1.0)	3.4 (0.0)

## Data Availability

Data available on request.

## Supplementary Materials

The following supporting information can be downloaded at: https://www.mdpi.com/article/10.3390/ma15238410/s1, Figure S1: Undecayed reference surfaces (a–f) of spruce; Figure S2: Undecayed reference surfaces (a–f) of birch; Figure S3: Images showing the fungal hyphae colonizing the modified surfaces of samples BF-CP (a) and SF-TV (b); Figure S4: Reference sample surfaces (a–c); Table S1: Composition data for reference and decayed samples.
