# Supplementary material for: Decay Resistance of Surface Carbonized Wood
Source: Materials (Basel). 2022 Nov 25;15(23):8410. doi: 10.3390/ma15238410 (PMC9737049; doi:10.3390/ma15238410)
Supplement: Supplementary file 1 [file materials-15-08410-s001.zip › materials-2038389-supplementary.pdf]

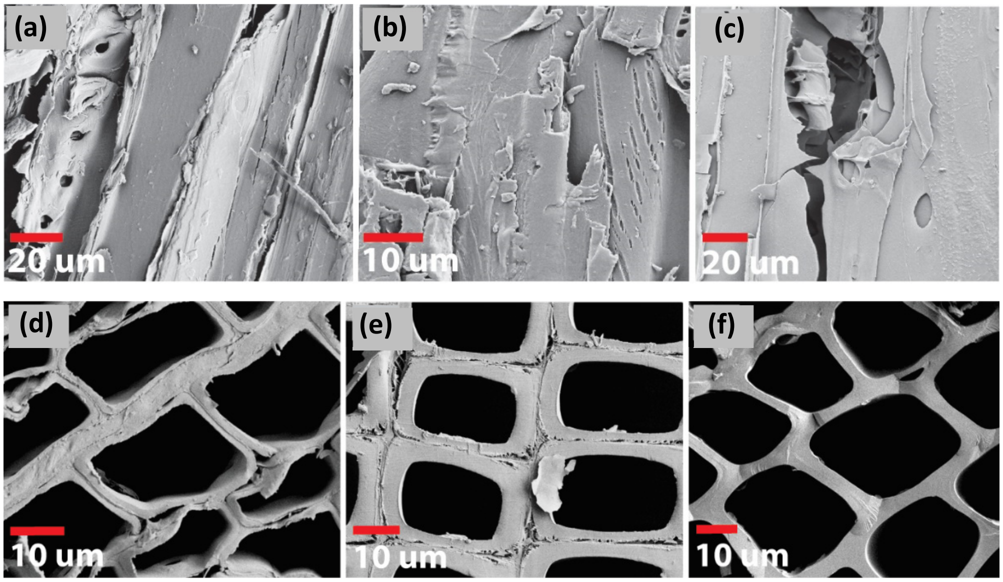

Figure S1. Undecayed reference surfaces (**a**–**f**) of spruce.

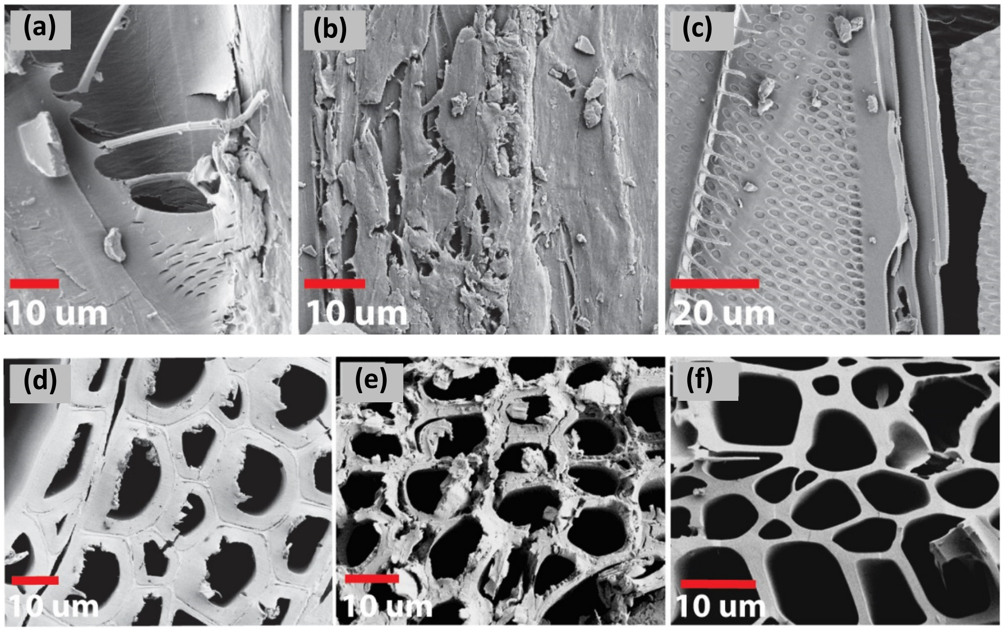

Figure S2. Undecayed reference surfaces (**a**–**f**) of birch.

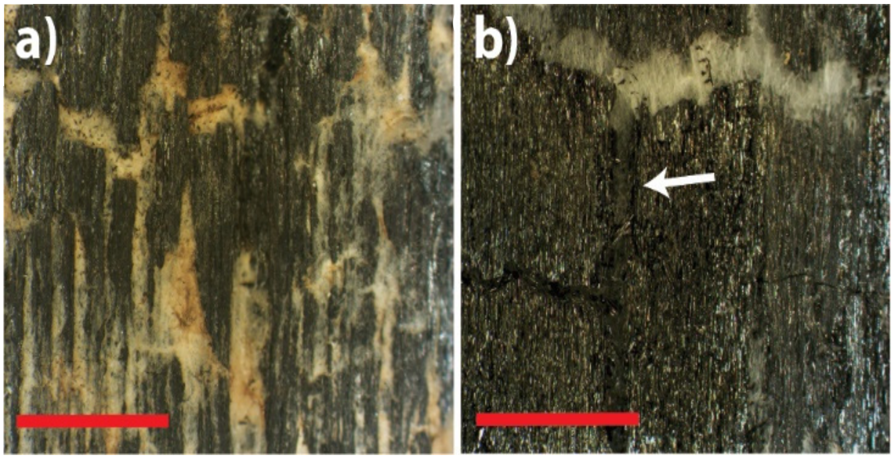

Figure S3. Images showing the fungal hyphae colonizing the modified surfaces of samples BF-CP (**a**) and SF-TV (**b**).

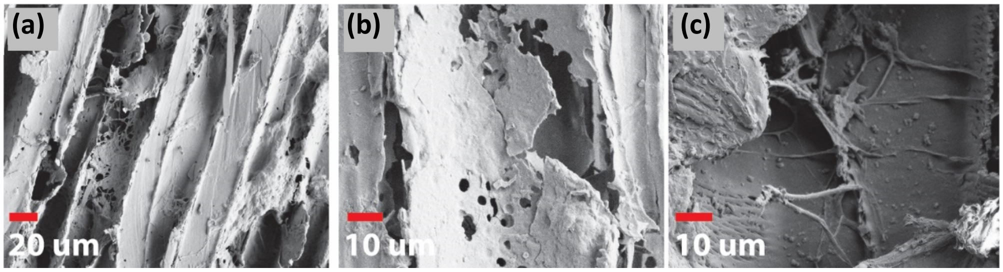

Figure S4. Reference sample surfaces (**a–c**).

Table S1. Composition data for reference and decayed samples.

Sample (Spruce)	Acid Soluble Lignin, (%)	Klason Lignin, %	Arabinose (%)	Rhamnose (%)	Galactose (%)	Glucose (%)	Xylose (%)	Mannose (%)
SR_0	0.49	27.25	1.14	0.25	3.85	48.28	5.99	13.12
SR-CP_0	1.92	58.35	0.81	0.44	1.82	14.98	3.26	1.74
SR-CP_2	1.59	59.31	0.56	0.18	1.90	15.41	2.98	1.83
SR-TV_0	1.80	31.42	1.21	0.31	4.14	42.97	5.78	11.44
SR-TV_2	1.22	37.42	1.21	0.26	3.91	44.00	5.86	11.61
SC_0	0.48	60.96	0.04	0.00	0.05	37.25	0.32	1.12
SC_2	0.64	30.62	0.69	0.12	1.62	49.66	5.33	11.72
SC-CP_0	0.78	56.75	0.07	0.00	0.31	41.75	0.74	2.85
SC-CP_2	0.98	30.02	0.41	0.05	1.51	45.36	3.33	8.15
SC-TV_0	0.75	51.78	0.11	0.00	0.55	44.26	1.06	3.48
SC-TV_2	1.11	31.14	0.50	0.12	2.07	49.29	3.85	9.65
SF_0	0.08	91.90	0.00	0.01	0.01	7.30	0.01	0.01
SF_2	0.47	28.49	1.17	0.27	3.45	50.68	6.04	14.06
SF-CP_0	1.01	49.88	0.44	0.05	1.08	45.64	3.01	5.97
SF-CP_2	1.66	47.58	0.60	0.12	0.93	25.20	3.40	3.56
SF-TV_0	1.75	28.65	0.59	0.07	1.76	46.95	4.15	8.97
SF-TV_2	1.07	22.73	1.16	0.23	2.54	49.11	6.22	11.89

Sample (Birch)	Acid Soluble Lignin,, (%)	Klason Lignin, %	Arabinose (%)	Rhamnose (%)	Galactose (%)	Glucose (%)	Xylose (%)	Mannose (%)
BR_0	4.01	17.76	0.53	0.60	0.93	48.36	28.76	0.99
BR-CP_0	4.64	36.13	0.21	0.36	0.39	29.75	17.45	0.84
BR-CP_2	4.86	34.96	0.24	0.37	0.38	32.21	18.39	0.92
BR-TV_0	5.16	25.42	0.43	0.47	0.81	36.37	19.05	1.89
BR-TV_2	5.93	28.20	0.57	0.58	0.99	34.88	21.09	1.41
BC_0	0.67	49.43	0.00	0.00	0.00	49.26	1.57	0.31
BC_2	3.26	20.93	0.24	0.33	0.53	46.43	23.71	1.65
BC-CP_0	1.06	48.87	0.01	0.00	0.07	46.52	2.15	0.40
BC-CP_2	2.16	29.65	0.11	0.13	0.29	50.13	14.71	1.23
BC-TV_0	1.21	46.70	0.04	0.02	0.15	52.68	5.22	0.60

BC-TV_2	2.64	29.22	0.14	0.20	0.36	55.40	18.67	1.56
BF_0	0.09	95.99	0.01	0.01	0.01	4.45	0.02	0.01
BF_2	3.95	18.32	0.51	0.63	1.06	48.72	28.01	0.82
BF-CP_0	1.83	53.26	0.11	0.06	0.25	43.74	6.49	0.65
BF-CP_2	4.71	43.62	0.23	0.33	0.43	25.08	12.99	0.31
BF-TV_0	2.35	42.72	0.16	0.21	0.49	44.24	10.50	0.69
BF-TV_2	5.02	23.84	0.66	0.56	1.07	37.57	24.15	0.70
